# Proteomic Characterization of Annexin l (ANX1) and Heat Shock Protein 27 (HSP27) as Biomarkers for Invasive Hepatocellular Carcinoma Cells

**DOI:** 10.1371/journal.pone.0139232

**Published:** 2015-10-02

**Authors:** Ruo-Chiau Wang, Chien-Yu Huang, Tai-Long Pan, Wei-Yu Chen, Chun-Te Ho, Tsan-Zon Liu, Yu-Jia Chang

**Affiliations:** 1 Tissue Bank, Chang Gung Memorial Hospital, Chiayi, Taiwan; 2 Division of General Surgery, Department of Surgery, Shuang Ho Hospital, Taipei Medical University, Taipei, Taiwan; 3 Department of Neurosurgery, Shuang Ho Hospital, Taipei Medical University, Taipei, Taiwan; 4 School of Traditional Chinese Medicine, Chang Gung University, Taoyuan, Taiwan; 5 Research Center for Industry of Human Ecology, Chang Gung University of Science and Technology, Taoyuan, Taiwan; 6 Liver Research Center, Chang Gung Memorial Hospital, Taoyuan, Taiwan; 7 Graduate Institute of Clinical Medicine, Taipei Medical University, Taipei, Taiwan; 8 Department of Pathology, School of Medicine, College of Medicine, Taipei Medical University, Taipei, Taiwan; 9 Department of Pathology, Wan Fang Hospital, Taipei Medical University, Taipei, Taiwan; 10 Graduate Institute of Medical Science, Taipei Medical University, Taipei, Taiwan; 11 Translational Research Laboratory, Cancer Center, Taipei Medical University Hospital, Taipei, Taiwan; 12 Division of General Surgery, Department of Surgery, Taipei Medical University Hospital, Taipei Medical University, Taipei, Taiwan; 13 Cancer Research Center, Taipei Medical University Hospital, Taipei Medical University, Taipei, Taiwan; The University of Hong Kong, HONG KONG

## Abstract

To search for reliable biomarkers and drug targets for management of hepatocellular carcinoma (HCC), we performed a global proteomic analysis of a pair of HCC cell lines with distinct differentiation statuses using 2-DE coupled with MALDI-TOF MS. In total, 106 and 55 proteins were successfully identified from the total cell lysate and the cytosolic, nuclear and membrane fractions in well-differentiated (HepG2) and poorly differentiated (SK-Hep–1) HCC clonal variants, respectively. Among these proteins, nine spots corresponding to proteins differentially expressed between HCC cell types were selected and confirmed by immunofluorescence staining and western blotting. Notably, Annexin 1 (ANX1), ANX–2, vimentin and stress-associated proteins, such as GRP78, HSP75, HSC–70, protein disulfide isomerase (PDI), and heat shock protein–27 (HSP27), were exclusively up-regulated in SK-Hep–1 cells. Elevated levels of ANX–4 and antioxidant/metabolic enzymes, such as MnSOD, peroxiredoxin, NADP-dependent isocitrate dehydrogenase, α-enolase and UDP-glucose dehydrogenase, were observed in HepG2 cells. We functionally demonstrated that ANX1 and HSP27 were abundantly overexpressed only in highly invasive types of HCC cells, such as Mahlavu and SK-Hep–1. Knockdown of ANX1 or HSP27 in HCC cells resulted in a severe reduction in cell migration. The in-vitro observations of ANX1 and HSP27 expressions in HCC sample was demonstrated by immunohistochemical stains performed on HCC tissue microarrays. Poorly differentiated HCC tended to have stronger ANX1 and HSP27 expressions than well-differentiated or moderately differentiated HCC. Collectively, our findings suggest that ANX1 and HSP27 are two novel biomarkers for predicting invasive HCC phenotypes and could serve as potential treatment targets.

## Introduction

Hepatocellular carcinoma (HCC) is one of the most common malignancies in the world, with a mortality rate of approximately one million each year [1, 2]. The prognosis of HCC remains poor even with a combination of chemotherapies and radiation therapies because of intrinsic and/or acquired treatment resistance and a high rate of metastasis [3, 4]. Thus, a better understanding of the biochemical and molecular properties of HCC may lead to the development of biomarkers and therapeutic strategies. Differentiation is an important cellular process that regulates the clonal increase of the cell population, and the differentiation status of a cancer cell is known to play a pivotal role in the extent of carcinogenesis and its metastatic propensity [5]. Thus, the identification of molecules that determine the differentiation status (i.e., mesenchymal or epithelial) of HCC may provide important clues for drug development.

The differentiation of human hepatocytes is particularly interesting because varieties of plasma protein markers have been well characterized [6–8]. Because HCC is a hepatocyte malignancy, Chang et al. previously proposed that the expression patterns of plasma proteins and/or plasma membrane protein markers could be used as an approach for studying human HCC differentiation status [9]. However, this technique, although specific, is laborious and time-consuming because of the necessity of analyzing at least 15 different plasma proteins secreted in the culture medium. Subsequently, some independent differentiation-associated biomarkers have been discovered [10–13], but their clinical significance has not been verified thus far.

The current interest in proteomics has arisen in part because of the prospect that a proteomic approach to disease investigation may overcome some of the limitations encountered by other methodologies [14, 15]. With this premise in mind, we aimed to identify protein biomarkers in different components of HCC cells with distinct disparities in differentiation status. The rationale for this approach is that protein expression during cell differentiation may vary among different compartments (cytosol, nucleus and membrane fractions) of HCC cells. Some of these proteins may play pivotal roles in controlling the proliferative capability and metastatic behaviors. Furthermore, the translocation of proteins to the nucleus may also be crucial in initiating various biological events. In this study, we examined the protein expression in different cellular compartments and identified candidate proteins that were overexpressed or down-regulated in two HCC cell lines with distinct differentiation states. The identified proteins and their proposed functions may provide important information for therapeutic designs and may serve as potential biomarkers for predicting disease progression or treatment responses.

## Materials and Methods

### Origin and characteristics of HCC cells used in this study

A panel of five HCC subline variants was selected for this study, and their differentiation statuses were established based on their morphological characteristics, secreted plasma protein profiles, pattern of lactate dehydrogenase (LD) isoenzyme expression[11], pattern of thyroid hormone β1 nuclear receptor (h-TRβ1) expression and pattern of hepatocyte-derived growth factor (HDGF) expression [16]. The HepG2 subline, a well-differentiated HCC variant, and the SK-Hep–1 subline, a poorly-differentiated HCC variant, were selected for proteomic analysis.

### Cell culture

HepG2, Hep3B, and SK-Hep–1 cells were purchased from ATCC. HepJ5 and Mahlavu cell were gifted from Dr. C.S Yang, National Taiwan University and Dr. C. P. Hu, Veterans General Hospital, Taiwan [17, 18]. Those cells were cultured in Dulbecco’s modified Eagle medium (DMEM) (Sigma Chemical Co., St. Louis. MO, USA) supplemented with 10% fetal bovine serum (FBS) (Hyclone Laboratories, Logan, UT, USA) and incubated at 37°C in a humidified atmosphere with 5% CO_2_.

### Two-dimensional electrophoresis

Cultured cells were solubilized in lysis buffer containing 7 M urea, 2 M thiourea, 4% CHAPS, 1% IPG buffer, pH3-10 (GE Healthcare Life Sciences, Piscataway, NJ, USA), 65 mM DTT, and 10 nM PMSF. The protein concentration was measured using the Bradford assay (Bio-Rad, Hercules, CA, USA). Proteins were applied onto isofocusing gels (13-cm IPG linear strip, GE Healthcare Life Sciences, Piscataway, NJ, USA). The running conditions for the IEF were as follows: 30 V for 12 h, 100 V for 0.5 h, 250 V for 0.5 h, 500 V for 0.5 h, 1000 V for 0.5 h, 2000 V for 0.5 h, 4000 V for 0.5 h, and 8000 V for 0.5 h, up to 70 kV-h. IPG strips were equilibrated for 10 min in a solution containing 50 mM Tris-HCl (pH8.8), 6 M urea, 20% SDS, 30% glycerol, 2% DTT, and a trace of bromophenol blue, followed by 10 min in the same solution except that DTT was replaced with 2.5% iodoacetamide. The IPG gel strips were embedded in gels containing 0.5% agarose. 2-D SDS-PAGE was performed on 12% acrylamide gels (GE Healthcare, USA) at 6 mA/gel until the bromophenol blue dye front reached the bottom of the gel. After approximately 16–18 h, all gels were visualized using a mass-compatible silver stain and scanned using an image scanner (GE Healthcare, USA). Protein spots were quantified using Nonlinear Progenesis software (technical support provided by J&H Technology, Taipei, Taiwan). All experiments were repeated at least three times.

### Tryptic in-gel digestion of proteins and MALDI-TOF MS

Selected protein targets of approximately 1 mm in diameter on the 2-D gels were manually excised. Spots excised from the stained gels were processed according to the standard MS sample preparation protocol [19, 20]. In-gel digestion of proteins was performed using MS-grade Trypsin Gold (Promega, Madison, WI) overnight at 37°C. Tryptic digests were extracted using 10 μL Milli-Q water initially, followed by two extractions with a total of 20 μL of 50% acetonitrile / 0.1% trifluoroacetic acid. The combined extracts were dried in a vacuum concentrator at room temperature and dissolved in 1 μL of 5% acetonitrile with 0.5% trifluoroacetic acid. The ESI-MS/MS mass spectrometer utilized for protein analysis was a Thermo LTQ-Orbitrap (Thermo Scientific, UK). The MS/MS signal was analyzed using the MASCOT search engine (www.matrixscience.com). The search parameters were defined as follows: database, NCBInr 20120129; taxonomy, viridiplantae (Green Plants); enzyme, trypsin; fixed modification, carbamidomethylation; variable modifications, oxidation; peptide MS tolerance, ± 0.5 Da; and fragment MS tolerance, ± 0.5 Da and allowance of one missed cleavage.

### Separation of proteins from HCC compartments and western blotting

Subcellular fractions were prepared using the Pierce Cytoplasmic Nuclear extraction kit (Thermo, USA). In brief, harvested cells were resuspended with CERI and CERII and centrifuged at 16000x g for 5 min, and the cytoplasmic fraction (supernatant) was separated from the nuclei (pellet). Subsequently, the pellet was resuspended with NER. After centrifugation at 16000x g for 10 min, the supernatant was collected as the nuclear fraction. 2-DE lysis buffer with 8 M urea and 4% CHAPS was added to the remaining pellet to isolate the membrane fraction. The protein samples were separated using SDS-PAGE and analyzed via immunoblotting. The primary antibodies used targeted vimentin, Annexin1 (ANX1), ANX4, and heat shock protein–27 (HSP27) (1:1000; Santa Cruz Biotech., Santa Cruz, CA, USA). Enhanced chemiluminescence (Immobilon Western Chemiluminescent AP substrate or ECL reagent, Millipore, Billerica, MA, USA) was used for detection. Expression of β-actin was used to control for equal gel loading.

### Immunofluorescence staining

Approximately 1×10^5^ cells were seeded in 6-well culture plates. When the cells were 50–60% confluent, they were fixed in an iced acetone and methanol 1:1 (v/v) mixed solution for 15 min. The cells were permeabilized with PBS containing 1% (v/v) Triton X–100 and incubated with primary antibodies. To detect primary antibody binding sites, the plates were washed and stained with FITC-conjugated secondary antibodies. After washing in PBS, propidium iodide (PI) was added as a nuclear counterstain. The cells were visualized using a fluorescence microscope (Nikon).

### Gene silencing

Expression of ANX1 and HSP27 in HCC cells was ablated using Mission shRNA clones from Sigma Chemical Co. (St. Louis, MO). Mission shRNA clones are sequence-verified shRNA lentiviral plasmids purchased from the National RNAi Core Facility (Taiwan, ROC) for gene silencing in mammalian cells. The parental vector (pLKO.1<-puro) enables transient transfection or stable selection through puromycin resistance. The target sequence for the human ANXA1 mRNA (NM_000700.1) gene was 5’-CATAAGGCCATAATGGTTAAA–3’. The target sequence for the human HSP27 mRNA (NM_001540.3) gene was 5’-CCGATGAGACTGCCGCCAAGT–3’. The non-target shRNA control vector (SHC002) was purchased from Sigma Chemical Co. (St. Louis, MO), and the sequence of scrambled shRNA was 5’-CAACAAGATGAAGAGCACCAA–3’. Briefly, 1.5×10^5^ cells were washed twice with PBS and mixed with 0.5 μg of the plasmid. One pulse was applied for 20 ms under a fixed voltage of 1.4 kV on a pipette-type Neon microporator (Life Technologies). Stably transfected cells were selected by puromycin for 2 weeks. The expression level of ANX1 and HSP27 was determined by real-time PCR and western blotting.

### Transwell migration assay

In vitro cell migration studies were performed using a BD Falcon cell culture insert (BD Biosciences, Durham, NC) as previously described [21]. Briefly, we suspended 1×10^5^ cells in 500 μL of serum-free DMEM, and the cells were seeded into the upper part of each chamber. The lower compartment of each chamber was filled with 1 mL of DMEM that contained a 10% FCS serum gradient. After incubation for 24 hours at 37°C in 5% CO_2_, the non-migrating cells were removed from the upper surface of the membrane by scraping. Cells on the reverse side of the membrane were stained with 0.1% crystal violet. The migrating cells were counted under a microscope at 100-fold magnification.

### Real-time quantitative reverse transcription-PCR (qRT-PCR) analysis

Total RNA was isolated from colon cancer cells using Trizol reagent according to the manufacturer’s instructions (Invitrogen Life Technologies). The cDNA was amplified from 2 μg of total RNA in a final volume of 20 μL using Moloney murine leukemia virus (M-MLV) reverse transcriptase at 37°C for 90 min. The sequences of the qPCR primers were 5’-CCCCCATGCCCAAGCTA -3’ (forward) and 5’-TCGAAGGTGACTGGGATGGT -3’ (reverse) for HSP27, 5’-AAAGGTGGTCCCGGATCAG -3’ (forward) and 5’-CATCCACACCTTTAACCATTATgg -3’ (reverse) for ANX1, 5'-AGCGCGGCTACAGCT–3'(forward) and 5’-GGCCATCTCTTGCTCGAAGT–3’ (reverse) for β-actin. The quantitative RT-PCR reaction was performed using ABI SYBR Green Master Mix using in an ABI StepOne system (Applied Biosystems, Grand Island, NY). Thermal cycling was performed in the ABI StepOne system. The quantitative PCR conditions were 95°C for 10 min followed by 40 cycles of 95°C for 15 s and 60°C for 1 min. The cycle parameters were 95°C for 10 minutes, followed by 40 cycles of 95°C for 15 seconds, 60°C for 1 minute, and a final extension at 72°C for 10 minutes.

### Tissue microarray staining by HSP27 and ANX1

Tissue microarray sets totally including 80 cases of primary HCC (catalog No. CSA4 and CS4) were purchased from SuperBioChips Laboratories. (Seoul, South Korea). The pathologic diagnosis and tumor grading of these cases were microscopically reconfirmed by a pathologist. The grading system of World Health Organization was used. HCC was divided into well-differentiated (WD), moderately differentiated (MD) and poorly differentiated (PD). An immunohistochemical stain with HSP27 (catalog No.: ab2790. 1:750; Abcamplc, Cambridge, United Kingdom) was performed. Expression levels of HSP27 were scored semiquantitatively as weakly positive (1+), moderately positive (2+) and strongly positive (3+). To understand the correlation between pathologic characteristics and expression levels of ANX1, two tissue microarray sets totally including 88 cases of HCC (catalog No. CSA4 and CS5) were purchased from SuperBioChips Laboratories. An immunohistochemical stain with ANX1 (clone: MRG3. 1:150; Cell Marque Corp., Rocklin, California, USA) was performed. Expression levels of ANX1 were scored semiquantitatively as negative, weakly positive and strongly positive. The association between pathologic characteristics and ANX1 expression was analyzed.

### Statistical analysis

All experiments were repeated a minimum of three times. All data collected from real-time RT-PCR analysis, MTT assays and migration assays are expressed as the mean ± SD. The data presented in some figures are from a single experiment that was quantitatively similar to the replicate experiments. Statistical significance was determined using Student’s t test (two-tailed) or chi-square test to compare two groups of data sets.

## Results

### Identification of abundantly expressed proteins in HCC cells

**Fig 1:**
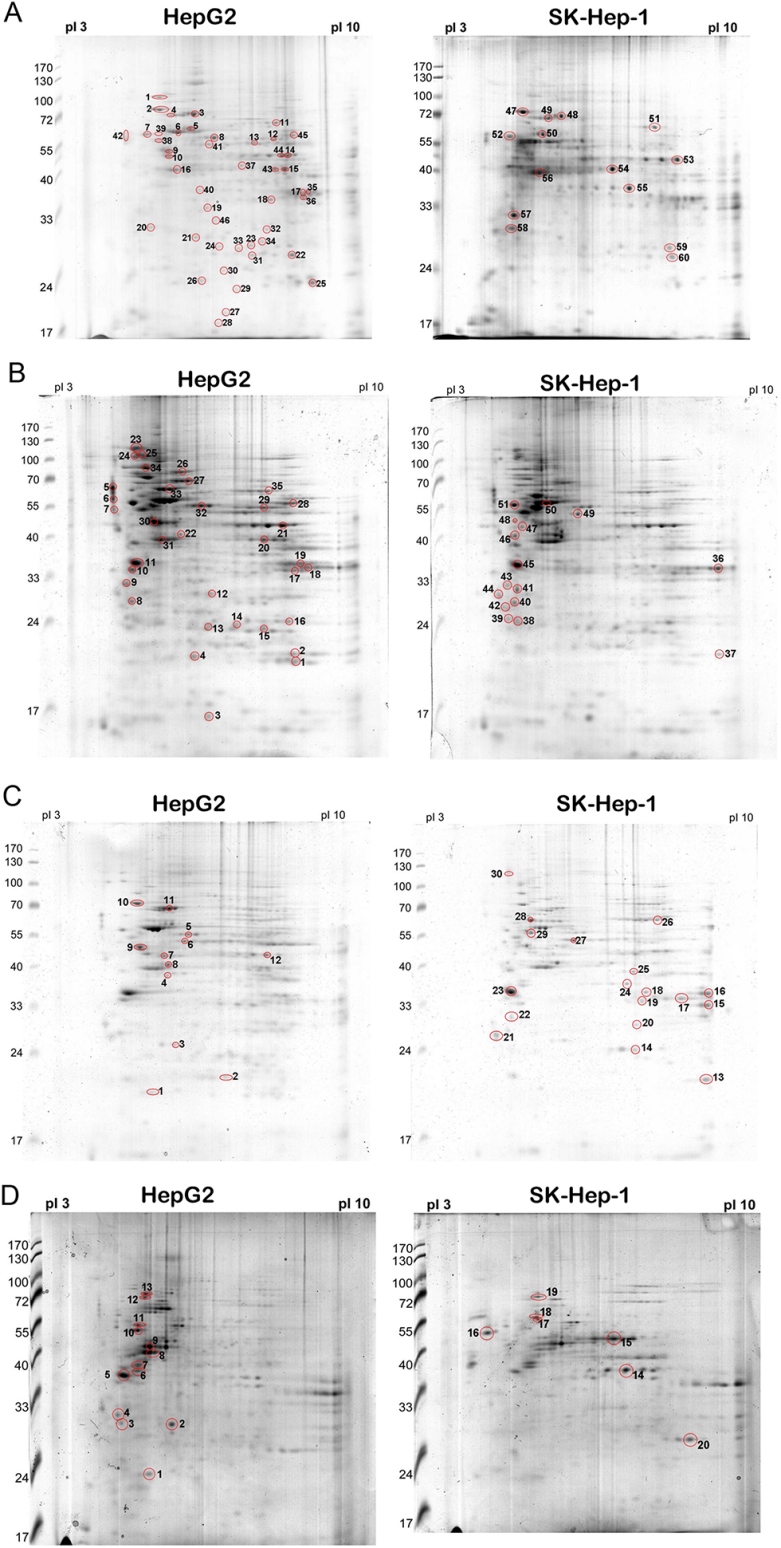
Representative 2D gel images of various cellular compartments depicting identified protein spots that were differentially expressed between HepG2 and SK-Hep–1 clonal variants. (A) Total cell lysate, (B) cytosol, (C) nucleus, and (D) membrane. Differentially expressed proteins are numbered and boxed. Reference proteins are indicated by arrowheads.

**Fig 2:**
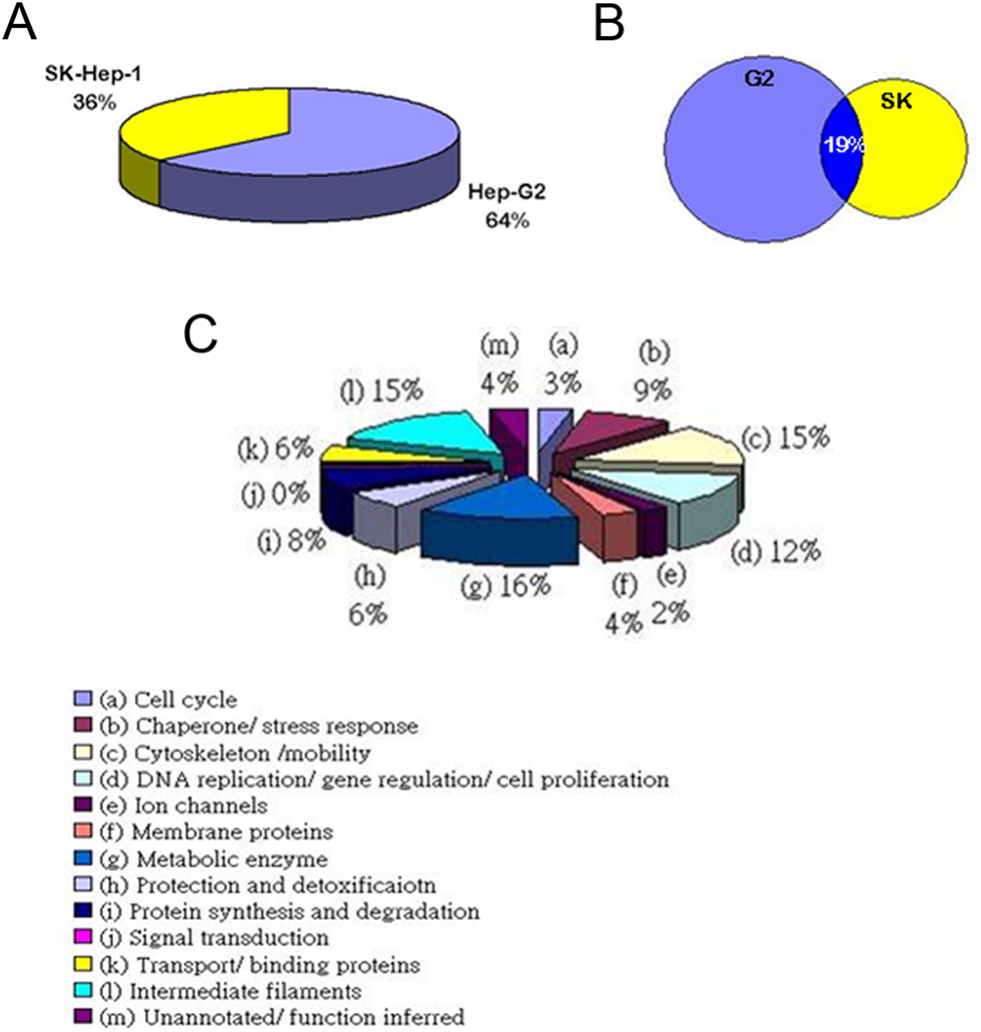
Protein classification. The functional classifications of the identified proteins.a: Cell cycle: 3%; b: chaperone/stress response: 9%; c: cytoskeleton/ cell mobility: 15%; d: DNA replication/gene regulation/cell proliferation: 12%; e: ion channels: 2%; f: membrane proteins: 4%; g: metabolic enzyme: 16%; h: protection and detoxification: 6%; i: protein synthesis and degradation: 8%; j: signal transduction: 8%; k: transport/binding proteins: 6%; l: intermediate filaments: 15%; and m: unannotated/ function inferred: 4%.

Approximately 450 μg of the total protein obtained from total cell lysates of either HepG2 or SK-Hep–1 cells was focused by IEF on pH 3–10 nonlinear IPG strips before being separated on a 12% polyacrylamide gel (PAGE). The spots were visualized by Coomassie blue G250 staining. Among the 60 spots that were identified, 46 were associated with HepG2 cells, and 14 spots belonged to SK-Hep–1 cells. Protein spots of interest were analyzed using MALDI-TOF and the MALDI spectra followed by identification using the MASCOT search engine. The MALDI-TOF identification of the protein spots from the lysates of HCC sublines is presented in Fig 1A and Table 1. We also isolated proteins from various cellular compartments of HepG2 and SK-Hep–1 cells. Cytosolic (500 μg), nuclear (250 μg) and membrane (250 μg) proteins were then subjected to IEF followed by PAGE. Protein spots were identified by MALDI-TOF, as indicated in **Fig 1B, 1C and 1D** and **Tables 2, 3 and 4**. Among the abundantly expressed protein spots on the 2-DE map (161 spots), a total of 106 spots (64%) belonged to the well-differentiated HepG2 cells, with the following order of distribution in each cellular compartment: total cell lysate (46; 46%)>cytosol (35; 33%)>membrane (13; 12%)>nucleus (12; 11%) (**Fig 2 and Tables 2, 3 and 4**). Conversely, a total of 55 spots (36%) belonged to the poorly differentiated SK-Hep–1 cells, with the following order of abundance in each cellular compartment: nucleus (18; 33%)>cytosol (16; 29%)>total cell lysate (14; 25%)>membrane (7; 13%) (**Fig 2 and Tables 2, 3 and 4**). Notably, the well-differentiated HCC cells overexpressed antioxidant/metabolic enzymes, such as MnSOD, peroxiredoxin (Prdx), NADP-dependent isocitrate dehydrogenase (ICDH), α-enolase and UDP-glucose dehydrogenase. In contrast, poorly differentiated HCC cells exhibited high levels of stress-associated proteins, such as Grp78, HSP75, HSC–70, protein disulfide isomerase (PDI), and HSP27 (**Tables 5 and 6**).

**Table 1:** Total proteins of HepG2 and SK-Hep–1 cells were identified by MALDI-TOF.

**Cells**	**Spot NO.**	**Accession NO.**	**Identification**	**Theoretical M.W./ PI**	**Score (Seq Cov)**	**Cellular localization**	**Molecular function**	**Class.**
**HepG2**								
	1		Dnak-type molecular chaperone HSPA5	72.185/5.03	188(61%)		Chaperone	b
	2		Dnak-type molecular chaperone HSPA5	72.185/5.03	256(62%)		Chaperone	b
	3		Dnak-type chaperone precursor	73.92/5.87	200(61%)		Chaperone	b
	4		Dnak-type chaperone precursor	71.082/5.37	141(56%)		Chaperone	b
	5	P61978	Heterogeneous nuclear ribonucleoprotein K	47.756/5.46	102(46%)	Nucleus/ cytoplasm	Regulation of transcription	d
	6	P10809	HSP 60A	61.157/5.70	188(71%)	Mitochondrial matrix	Transport the mitochondrial proteins	b
	7	NP 00684	Protein disulfide-isomerase (ER60)	57.48/4.76	179(64%)	ER lumen	Folding of proteins containing disulfide bonds in ER	h, i
	8	NP 00684	Protein disulfide-isomerase (ER60)	57.146/5.98	189(60%)	ER lumen	Folding of proteins containing disulfide bonds in ER	i
	9	NP 057078	H^+^-transporting two-sector ATPase	56.525/5.26	145(77%)	Mitochondrion	ATP synthesis coupled proton transport	c, l
	10	P17980	Proteasome subunit p50	49.458/5.13	88(60%)	Nucleus/ cytoplasm	Ubiquitin-dependent Protein catabolism	i
**Cells**	**Spot NO.**	**Accession NO.**	**Identification**	**Theoretical M.W./ PI**	**Score (Seq Cov)**	**Cellular localization**	**Molecular function**	**Class.**
**HepG2**	11	P31948	Stress induced phosphoprotein1 (STI 1)	65.089/6.27	139(66%)	Nucleus/ cytoplasm	Modulation the molecular chaperones HSC 70 and HSP 90	b
	12	NP 000680	Aldehyde dehydrogenase 1A1	55.454/6.30	106(67%)	Cytoplasm	Alcohol metabolic enzyme	g
	13	P78371	T complex protein 1 subunit beta	57.794/6.01	185(79%)	Cytoplasm	Chaperone	b
	14	P06733	α-enolase	47.35/6.99	230(76%)	Cytoplasm	Metabolic enzyme (Glycolysis)	g
	15	NP 005887	NADP-dependent isocitrate dehydrogenase	46.944/6.34	149(75%)	Mitochondrial matrix	Metabolic enzyme (TCA cycle)	g
	16	NP 001606	Actin-gamma	42.108/5.31	124(82%)	Cytoplasm	Cytoskeleton, cell motility	c, l
	17	P42330	Aldo-keto reductase	37.23/8.06	81(61%)	Cytoplasm	Aldehyde metabolism	g
	18	P42330	Aldo-keto reductase family 1	36.20/6.76	62(61%)	Cytoplasm	Aldehyde metabolism	g
	19	P50225	Aryl sulfotransferase	34.288/5.68	127(84%)	Cytoplasm	Amine biosynthesis	g
	20	P07951	Tropomyosin	28.89/4.76	52(20%)	Cytoplasm	Cytoskeleton, regulation of muscle contraction	g
	21	P35232	Prohibitin	29.589/5.57	214(83%)	Mitochondria inner membrane	Gene regulation and modulate the cell proliferation	d
	22	P60174	Triosephosphate isomerase	26.807/6.51	127(84%)		Metabolic enzyme (Glycolysis)	g
	23		ER lumenal 28	29.032/6.77	80(61%)			m
	24		ER lumenal 28	29.032/6.77	75(57%)			m
	25	P30086	PEBP	21.027/7.42	81(81%)	Cytoplasm	Regulation of central nervous system	k
**Cells**	**Spot NO.**	**Accession NO.**	**Identification**	**Theoretical M.W./ PI**	**Score (Seq Cov)**	**Cellular localization**	**Molecular function**	**Class.**
**HepG2**	26	P32119	Peroxiredoxin II	21.92/5.67	74(49%)	Cytoplasm	Antioxidant enzyme	h
	27	NP 000260	NM23-H1	19.869/5.42	113(60%)	Nucleus/ cytoplasm	Modulate the cell proliferation and nucleotide metabolism	c, d
	28		ND					
	29	P52945	Insulin activator factor	83.906/5.6	69(20%)	Nucleus	Activate insulin	g
	30		ND					
	31	P60174	Triosephosphate isomerase (TIM)	26.807/6.51	150(76%)	Cytoplasm	Metabolic enzyme (Glycolysis)	g
	32	P25786	Proteasome (macropain), α-type 1	29.864/6.15	86(40%)	Nucleus/ cytoplasm	Ubiquitin-dependent protein catabolism	i
	33		Enoyl-CoA hydratase	31.807/8.34	167(73%)			m
	34	NP 002119	High-mobility group box 1	18.470/9.72	72(44%)	Nucleus	Gene regulation	d, h
	35	P52895	3-α-hydroxysteroid/ dihydrodiol dehydrogenase	37.221/8.02	239(85%)	Cytoplasm	Steroid metabolism	g
	36	P42330	Aldo-keto reductase family 1	36.226/7.12	155(70%)	Cytoplasm	Aldehyde metabolism	g
	37		ND					
	38	P10809	GroEL (HSP 60)	61.187/5.70	139(54%)	Mitochondrial matrix	Transport the mitochondrial proteins	b
	39	P10809	GroEL (HSP 60)	61.187/5.70	139(54%)	Mitochondrial matrix	Transport the mitochondrial proteins	b
	40	NP 777480	TGF-β receptor interacting protein 1	36.878/5.38	103(57%)	Nucleus	Gene regulation	d
**Cells**	**Spot NO.**	**Accession NO.**	**Identification**	**Theoretical M.W./ PI**	**Score (Seq Cov)**	**Cellular localization**	**Molecular function**	**Class.**
**HepG2**	41	NP 002264	Keratin 8	41.083/4.94	159(72%)	Intermediate filament	Cytoskeleton organization and biogenesis	c
	42	P27797	Calreticulin variant	47.061/4.30	120(38%)	ER	Protein folding	i
	43	NP 005887	NADP-dependent isocitrate dehydrogenase	46.931/6.53	144(66%)	Mitochondrial matrix	Metabolic enzyme (TCA cycle)	g
	44	P06733	α-enolase	47.35/6.99	288(73%)	Cytoplasm	Metabolic enzyme (Glycolysis)	g
	45	NP 003350	UDP-glucose dehydrogenase	55.674/6.73	225(77%)	Cytoplasm	Metablolic enzyme (Glycosaminoglycan biosynthesis)	g
	46	P09525	Annexin A4	33.759/5.64	60(32%)	Mitochondrion	Calcium binding protein	e, f, l
**SK-Hep–1**								
	47	P11021	Grp 78	72.402/5.07	250(57%)	ER lumen	Protein folding in ER	i
	48	Q12931	HSP 75	74.02/5.97	274(64%)	Mitochondrion	Expression an ATPase activity	b
	49	P11142	Heat-shock cognate protein 70	71.082/5.37	119(53%)	Nucleus/ cytoplasm	Chaperone	b
	50	P10809	Chaperonin	61.187/5.70	212(77%)	Mitochondrial matrix	Transport the mitochondrial proteins	b
	51	P31948	Stress induced phosphoprotein1 (STI 1)	63.227/6.40	164(56%)	Nucleus/ cytoplasm	Modulation the molecular chaperones HSC 70 and HSP 90	b
	52	NP 00684	Protein disulfide isomerase (PDI)	57.48/4.76	208(65%)	ER lumen	Folding of proteins containing disulfide bonds in ER	h, i
**Cells**	**Spot NO.**	**Accession NO.**	**Identification**	**Theoretical M.W./ PI**	**Score (Seq Cov)**	**Cellular localization**	**Molecular function**	**Class.**
**SK-Hep–1**	53	P06733	α-enolase	47.35/6.99	219(72%)	Cytoplasm	Metabolic enzyme (Glycolysis)	g
	54	AAH01751	Chromosome 20 open reading frame 4	43.843/5.66	59(31%)	Cytoplasm	cell growth	d
	55	P33992	Replication licensing factor, MCM5	82.990/8.56	83(33%)	Nucleus	Initiation of DNA replication	d
	56	NP 001606	Actin-gamma	42.108/5.31	118(72%)	Cytoplasm	Cytoskeleton, cell motility	c, l
	57	P06753	Tropomyosin3	32.856/4.72	87(58%)	Cytoplasm	Cytoskeleton, regulation of muscle contraction	c, l
	58	P06753	Tropomyosin3	27.386/4.77	100(51%)	Cytoplasm	Cytoskeleton, regulation of muscle contraction	c, l
	59	P18669	Phosphoglycerate mutase B	28.90/6.67	141(81%)	Cytoplasm	Metabolic enzyme (Glycolysis)	g
	60	P60174	Triosephosphate isomerase (TIM)	26.807/6.51	112(82%)	Cytoplasm	Metabolic enzyme (Glycolysis)	g

ND: None detected.

**The functional classification (Class)** of identified proteins is shown a ~ m. a: cell cycle; b: chaperone/ stress response; c: cytoskeleton/ cell mobility; d: DNA replication/ gene regulation/ cell proliferation; e: ion channels; f: membrane proteins; g: metabolic enzyme; h: protection and detoxification; i: protein synthesis and degradation; j: signal transduction; k: transport/ binding proteins; l: intermediate filaments; m: unannotated/ function inferred.

**Table 2:** Cytosolic fraction proteins of Hep G2 and SK-Hep–1 cells were identified by MALDI-TOF.

**Cells**	**Spot NO.**	**Accession NO.**	**Identification**	**Theoretical M.W./ PI**	**Score (Seq Cov)**	**Cellular localization**	**Molecular function**	**Class.**
**HepG2**								
	1	NP 00067	Mn SOD	22.304/6.86	105(64%)	Mitochondrion	Antioxidant activity	h
	2	NP 859048	Peroxiredoxin I	14.054/6.25	85(73%)	Mitochondrion	Antioxidant activity	h
	3		ND					
	4	P32119	Peroxiredoxin II	21.918/5.67	106(62%)	Cytoplasm	Antioxidant activity	h
	5	P27797	Calreticulin variant	47.061/4.30	181(60%)	ER	Protein folding	i
	6	P27797	Calreticulin variant	47.061/4.30	248(67%)	ER	Protein folding	i
	7	P27797	Calreticulin variant	47.061/4.30	182(54%)	ER	Protein folding	i
	8	P06753	Tropomyosin	27.387/4.71	156(52%)	Cytoplasm	Cytoskeleton, regulation of muscle contraction	c, l
	9	P12004	PCNA	29.092/4.57	91(63%)	Nucleus	Cell cycle regulation	a, d
	10	P06748	Nucleophosmin, B23	31.090/4.71	68(40%)	Nucleus	Regulate cell proliferation	a
	11	P52895	3-α-hydroxysteroid/ dihydrodiol dehydrogenase	37.221/8.02	114(66%)	Cytoplasm	Steroid metabolism	g
	12	P09525	Annexin A4	35.957/5.85	151(67%)	Mitochondrion	Calcium binding protein	e, f, l
	13	P30041	Peroxiredoxin VI	25.002/6.02	92(61%)	Cytoplasm	Antioxidant activity	h
	14	P04792	HSP 27	22.427/7.83	99(63%)	Nucleus/ Cytoplasm	Protein folding	b
	15	P60174	Triosephosphate isomerase (TIM)	26.807/6.51	169(79%)	Cytoplasm	Metabolic enzyme (Glycolysis)	g
**Cells**	**Spot NO.**	**Accession NO.**	**Identification**	**Theoretical M.W./ PI**	**Score (Seq Cov)**	**Cellular localization**	**Molecular function**	**Class.**
**HepG2**	16	P18669	Phosphoglycerate mutase 1	28.769/6.75	153(79%)	Cytoplasm	Metabolic enzyme (Glycolysis)	g
	17	P42330	Aldo keto reductase	36.226/7.12	115(67%)	Cytoplasm	Aldehyde metabolism	g
	18	NP 002037	Glucose 3 phosphate dehydrogenase	36.202/8.26	112(64%)	Cytoplasm	Metabolic enzyme (Glycolysis)	g
	19	P52895	3-α-hydroxysteroid/ dihydrodiol dehydrogenase	37.221/8.02	114(66%)	Cytoplasm	Steroid metabolism	g
	20	NP 005887	Isocitrate dehydrogenase	46.944/6.34	147(72%)	Mitochondrial matrix	Metabolic enzyme (TCA cycle)	g
	21	P06733	α-enolase	47.35/6.99	288(73%)	Cytoplasm	Metabolic enzyme (Glycolysis)	g
	22	P05783	Cytokeratin 18	47.305/5.27	268(69%)	Cytoplasm	Cytoskeleton	c
	23	NP 003290	Tumor rejection antigen, gp96	92.567/4.77	244(55%)	Cytoplasm	Protein folding and transport	h
	24	P19338	Nucleolin	58.576/4.57	96(32%)	Nucleus	DNA/ RNA binding	d
	25	NP 003290	Tumor rejection antigen, gp96	92.567/4.77	244(55%)	Cytoplasm	Protein folding and transport	h
**Cells**	**Spot NO.**	**Accession NO.**	**Identification**	**Theoretical M.W./ PI**	**Score (Seq Cov)**	**Cellular localization**	**Molecular function**	**Class.**
**HepG2**	26	P38646	Grp 75	74.019/5.97	271(58%)	Mitochondrion	Cell proliferation and Cellular aging	d
	27	NP 004125	HSP 70k 9B (mortalin–2)	74.093/6.04	268(58%)	Cytoplasm	Anti-apoptosis, protein folding	b
	28	NP 003350	UDP-glucose dehydrogenase	55.674/6.73	298(75%)	Cytoplasm	Metablolic enzyme (Glycosaminoglycan biosynthesis)	g
	29	P00352	Retinal dehydrogenase (AL1A1)	55.323/6.29	112(53%)	Cytoplasm	Metabolic enzyme (retinoic acid biosynthesis)	g
	30	P60709	β-actin	42.08/5.37	87(66%)	Cytoskeleton	Cytoskeleton	c, l
	31	NP 001606	gamma-actin	42.108/5.31	165(77%)	Cytoplasm	Cytoskeleton, cell motility	c, l
	32	NP 00684	Protein disulfide-isomerase (ER 60)	57.146/5.98	301(67%)	ER lumen	Folding of proteins containing disulfide bonds in ER	h, i
	33	P10809	Chaperonin GroEL procursor	61.187/5.70	162(57%)	Mitochondrial matrix	Transport the mitochondrial proteins	b, k
	34	P11021	Grp 78	72.407/5.07	188(55%)	ER lumen	Protein folding in ER	k
	35	P31939	Bifunctional purine biosynthesis protein, PURH	64.938/6.39	268(74%)	Nucleus/ cytoplasm	Nucleic acid metabolism	g
**Cells**	**Spot NO.**	**Accession NO.**	**Identification**	**Theoretical M.W./ PI**	**Score (Seq Cov)**	**Cellular localization**	**Molecular function**	**Class.**
**SK-Hep–1**								
	36	P04406	Glyceraldehyde-3-phosphate dehydrogenase (GAPDH)	36.202/8.26	112(64%)	Cytoplasm	Metabolic enzyme (Glycolysis)	g
	37	P56876	Probable thioredoxin peroxidase (PAGA)	22.324/8.27	165(71%)	Nucleus/ cytoplasm	Antioxidant enzyme	h
	38	P63104	14.3.3 protein zeta/delta	30.10/4.72	149(58%)	Cytoplasm	Signal transduction	j
	39	P31947	14.3.3 protein sigma (Stratifin)	27.871/4.68	96(44%)	Cytoplasm	Cell proliferation, signal transduction	a, d, j
	40	P06753	Tropomyosin	27.387/4.71	120(50%)	Cytoplasm	Cytoskeleton, regulation of muscle contraction	c, l
	41	P07951	Tropomyosin	32.856/4.72	98(52%)	Cytoplasm	Cytoskeleton, regulation of muscle contraction	c, l
	42	P62258	14.3.3 protein epsilon	29.326/4.63	137(66%)	Cytoplasm	Signal transduction	j
	43	P12004	PCNA	29.092/4.57	91(63%)	Nucleus	Cell cycle regulation	a, d
	44	BAA82513	Pre-mRNA splicing factor SP2p32	31.287/4.74	73(61%)	Cytoplasm	mRNA processing	d
	45	P06748	B23	31.090/4.71	82(43%)	Nucleus	Regulate cell proliferation	a
	46	P08670	Vimentin	53.545/5.06	283(72%)	Cytoskeleton	Cytoskeleton, cell motility	c, f, l
	47	P08670	Vimentin	53.545/5.06	306(71%)	Cytoskeleton	Cytoskeleton, cell motility	c, f, l
	48	P08670	Vimentin	53.464/4.99	281(80%)	Cytoskeleton	Cytoskeleton, cell motility	c, f, l
	49	NP 002264	Keratin 8	30.802/5.02	177(83%)	Intermediate filament	Cytoskeleton organization and biogenesis	c
**Cells**	**Spot NO.**	**Accession NO.**	**Identification**	**Theoretical M.W./ PI**	**Score (Seq Cov)**	**Cellular localization**	**Molecular function**	**Class.**
**SK-Hep–1**	50	P10809	Chaperonin GroEL procursor	61.187/5.70	162(57%)	Mitochondrial matrix	Transport the mitochondrial proteins	b, k
	51	NP 00684	Protein disulfide-isomerase	57.480/4.76	267(69%)	ER lumen	Folding of proteins containing disulfide bonds in ER	h, i

ND: None detected.

**The functional classification (Class)** of identified proteins is shown a ~ m. a: cell cycle; b: chaperone/ stress response; c: cytoskeleton/ cell mobility; d: DNA replication/ gene regulation/ cell proliferation; e: ion channels; f: membrane proteins; g: metabolic enzyme; h: protection and detoxification; i: protein synthesis and degradation; j: signal transduction; k: transport/ binding proteins; l: intermediate filaments; m: unannotated/ function inferred.

**Table 3:** Nuclear fraction proteins of Hep G2 and SK-Hep–1 cells were identified by MALDI-TOF.

**Cells**	**Spot NO.**	**Accession NO.**	**Identification**	**Theoretical M.W./ PI**	**Score (Seq Cov)**	**Cellular localization**	**Molecular function**	**Class.**
**HepG2**								
	1	P56876	Probable thioredoxin peroxidase (PAGA)	22.324/8.27	165(71%)	Nucleus/ cytoplasm	Antioxidant enzyme	h
	2	NP 002119	High-mobility group box 1	18.470/9.72	48(52%)	Nucleus	Cell proliferation, anti-apoptosis	d, h
	3	NP 112533	Heterogeneous nuclear ribonucleoprotein B1	37.464/8.97	182(61%)	Nucleus	Regulation of transcription	d
	4	P04406	Glyceraldehyde-3-phosphate dehydrogenase (GAPDH)	36.07/8.58	98(58%)	Cytoplasm	Metabolic enzyme (Glycolysis)	g
	5	P07355	Annexin A2 (lipocortin II)	38.677/7.56	216(67%)	Plasma membrane	Calcium binding protein	e, f, l
	6	P04083	Annexin A1 (lipocortin1)	38.787/6.64	202(64%)	Plasma membrane	Calcium binding protein	e, f, l
	7	P07355	Annexin A2 (lipocortin II)	38.677/7.56	216(67%)	Plasma membrane	Calcium binding protein	e, f, l
	8	NP 003657	Basic leucine zipper nuclear factor 1 (JEM–1)	13.750/9.36	60(65%)	Nucleus/ cytoplasm	Cell proliferation and gene regulation	d
	9	P 24534	Elongation factor 1-β	24.788/4.50	56(41%)	Cytoplasm	Regulate translation	d
	10		ND					
**Cells**	**Spot NO.**	**Accession NO.**	**Identification**	**Theoretical M.W./ PI**	**Score (Seq Cov)**	**Cellular localization**	**Molecular function**	**Class.**
**HepG2**	11	P06748	Nucleophosmin, B23	29.617/4.47	69(38%)	Nucleus	Regulate cell proliferation	a
	12	P33991	DNA replication licensing factor, MCM4	97.068/6.28	43(13%)	Nucleus	Initiation of DNA replication	d
**SK-Hep–1**								
	13	NP 006146	Septin 2 (NEDD5)	36.824/6.85	74(33%)	Cytoplasm	GTP binding	a, d
	14	P02545	Lamin C	65.153/6.40	254(67%)	Nucleus	Protein binding	l
	15	NP 002264	Keratin 8	30.802/5.02	203(81%)	Intermediate filament	Cytoskeleton organization and biogenesis	c
	16	NP 001034267	ATP sythase D chain, mitochondria	18.405/5.22	62(53%)	Mitochondrion	ATP synthesis	g
	17	NP 031478	Peroxiredoxin III	28.047/7.11	63(52%)	Mitochondrion	Antioxidant activity	h
	18	P35232	Prohibitin	29.859/5.57	184(79%)	Mitochondrial inner membrane	Gene regulation and modulate the cell proliferation	d
	19	NP 038470	Somatin-like protein 2 (SLP–2)	38.642/6.88	132(69%)	Cytoskeleton	Receptor binding	c, f
	20	NP 00684	Protein disulfide-isomerase(ER 60)	57.146/5.98	301(67%)	ER lumen	Folding of proteins containing disulfide bonds in ER	h, i
**Cells**	**Spot NO.**	**Accession NO.**	**Identification**	**Theoretical M.W./ PI**	**Score (Seq Cov)**	**Cellular localization**	**Molecular function**	**Class.**
**SK-Hep–1**	21	NP 002264	Keratin 8 (fragment)	41.083/4.94	127(51%)	Intermediate filament	Cytoskeleton organization and biogenesis	c
	22	NP 002264	Keratin 8 (fragment)	41.083/4.94	127(51%)	Intermediate filament	Cytoskeleton organization and biogenesis	c
	23	P05783	Cytokeratin 18	47.305/5.27	268(69%)	Cytoplasm	Cytoskeleton	c
	24	NP 001677	H^+^-transporting two-sector ATPase, β chain	56.525/5.26	250(69%)	Mitochondrion	ATP synthesis coupled proton transport	k
	25		Dnak-type molecular chaperone precursor HSPA5 precursor	72.185/5.03	288(50%)		Chaperone	b
	26	P38646	Grp 75	74.019/5.97	355(63%)	Mitochondrion	Cell proliferation and Cellular aging	d
	27	P08670	Vimentin	53.545/5.06	306(71%)	Cytoskeleton	Cytoskeleton, cell motility	c, f, l
	28	P08670	Vimentin	53.545/5.06	306(71%)	Cytoskeleton	Cytoskeleton, cell motility	c, f, l
	29	P06733	α-enolase	47.35/6.99	128(58%)	Cytoplasm	Metabolic enzyme (Glycolysis)	g
	30	P11021	Grp 78	72.402/5.07	223(53%)	ER lumen	Protein folding in ER	i

ND: None detected.

**The functional classification (Class)** of identified proteins is shown a ~ m. a: cell cycle; b: chaperone/ stress response; c: cytoskeleton/ cell mobility; d: DNA replication/ gene regulation/ cell proliferation; e: ion channels; f: membrane proteins; g: metabolic enzyme; h: protection and detoxification; i: protein synthesis and degradation; j: signal transduction; k: transport/ binding proteins; l: intermediate filaments; m: unannotated/ function inferred.

**Table 4:** Membrane fraction proteins of Hep G2 and SK-Hep–1 cells were identified by MALDI-TOF.

**Cells**	**Spot NO.**	**Accession NO.**	**Identification**	**Theoretical M.W./ PI**	**Score (Seq Cov)**	**Cellular localization**	**Molecular function**	**Class.**
**HepG2**								
	1	NP 001034267	ATP synthase D chain	18.405/5.22	80(68%)	Mitochondrion	ATP synthesis	g
	2	P35232	Prohibitin	29.859/5.57	135(73%)	Mitochondrial inner membrane	Gene regulation and modulate the cell proliferation	c, l
	3	P06576	ATP synthase β-subunit	34.026/4.90	61(50%)	Mitochondrion	ATP synthesis	g
	4	P06753	Tropomyosin3	27.386/4.77	86(42%)	Cytoplasm	Cytoskeleton, regulation of muscle contraction	c, l
	5	P06748	Nucleophosmin, B23	33.026/4.6	54(34%)	Nucleus	Regulate cell proliferation	a
	6	NP 004491	Heterogeneous nuclear ribonucleoprotein C	27.861/4.55	117(59%)	Nucleus	mRNA processing	d, h
	7	P60709	Actin β subunit	40.536/5.55	63(40%)	Cytoskeleton	Cytoskeleton	c, l
	8	P60709	Actin β chain	41.321/5.56	114(58%)	Cytoskeleton	Cytoskeleton	c, l
	9	NP 001606	Actin-gamma	42.108/5.31	57(39%)	Cytoplasm	Cytoskeleton, cell motility	c, l
	10	NP 057078	H^+^-transporting two-sector ATPase	56.525/5.26	132(53%)	Mitochondrion	ATP synthesis coupled proton transport	k
	11	NP 001677	H^+^-transporting two-sector ATPase β chain	56.525/5.26	170(65%)	Mitochondrion	ATP synthesis coupled proton transport	k
**Cells**	**SpotNO.**	**Accession NO.**	**Identification**	**Theoretical M.W./ PI**	**Score (Seq Cov)**	**Cellular localization**	**Molecular function**	**Class.**
	12	P20100	Lamin B1	66.522/5.11	273(53%)	Nucleus	Structure protein	l
	13	P20100	Lamin B1	66.522/5.11	256(58%)	Nucleus	Structure protein	l
**SK-Hep–1**								
	14	AAK01919	mAb 3H11 antigen	69.954/5.19	66(24%)	Nucleus/ cytoplasm	Protein binding	k
	15		ND					
	16		ND					
	17	P08670	Vimentin	49.680/5.19	211(62%)	Cytoskeleton	Cytoskeleton, cell motility	c, f, l
	18	P08670	Vimentin	49.680/5.19	204(66%)	Cytoskeleton	Cytoskeleton, cell motility	c, f, l
	19	P20100	Lamin B1	66.522/5.11	186(54%)	Nucleus	Structure protein	l
	20	NP 112533	Heterogeneous nucleus ribonucleoprotein B1	37.464/8.97		Nucleus	mRNA processing	d, h

ND: None detected.

**The functional classification (Class)** of identified proteins is shown a ~ m. a: cell cycle; b: chaperone/ stress response; c: cytoskeleton/ cell mobility; d: DNA replication/ gene regulation/ cell proliferation; e: ion channels; f: membrane proteins; g: metabolic enzyme; h: protection and detoxification; i: protein synthesis and degradation; j: signal transduction; k: transport/ binding proteins; l: intermediate filaments; m: unannotated/ function inferred.

**Table 5:** List of unregulated proteins in the 2DE map of either the well-differentiated HepG2 cells or the poorly differentiated SK-Hep–1 cells identified by MALDI-TOF MS.

Cellular compartemts	Spot No	Association code	Name	Theoretical (kDa)/pI	HepG2	SK-Hep–1	Fold	Cellular localization	Function
**Total Cell lysate**	1	NP005887	NADP^+^-dependent isocitrate dehydrogenase	46.944/6.34	↑	↓	0.01	Mitochondria	Metabolic enzyme in TCA cycle
	2	P32119	Peroxiredoxin II	21.918/5.67	↑	↓	0.27	Cytoplasm	Antioxidant enzyme
	3	NP003350	UDP-glucose dehydrogenase	55.674/6.73	↑	↓	0.19	Cytoplasm	Metabolic enzyme in Glycosaminoglycan biosynthesis
	4	P09525	Annexin 4	33.757/5.64	↑	↓	0.34	Mitochondria	Calcium binding protein
	5	P33992	Replication licensing factor MCM5	82.990/8.56	↓	↑	4.09	Nucleus	Initiation of DNA replication
	6	P06753	Tropomyosin3	27.386/4.77	↓	↑	4.1	Cytoplasm	Cytoskeleton
**Cytosol**	1	P09525	Annexin 4	35.957/5.85	↑	↓	0.45	Mitochondria	Calcium binding protein
	2	NP005887	NADP+-dependent isocitrate dehydrogenase	46.944/6.34	↑	↓	0.01	Mitochondria	Metabolic enzyme in TAC cycle
	3	NP00067	MnSOD	22.304/6.86	↑	↓	0.2	Mitochondria	Antioxidant enzyme
	4	NP859048	Peroxiredoxin I	14.054/6.25	↑	↓	0.22	Mitochondria	Antioxidant enzyme
	5	P32119	Peroxiredoxin II	21.918/5.67	↓	↑	0.6	Cytoplasm	Antioxidant enzyme
	6	NP003350	UDP-glucose dehydrogenase	55.674/6.73	↑	↓	0.34	Cytoplasm	Glycosaminoglycan
	7	P04792	Heat Shock Protein 27	22.427/7.83	↓	↑	1.03	Nucleus/ Cytoplasm	Protein folding
	8	P08670	Vimentin	53.545/5.06	↓	↑	1.23	Cytoplasm	Cell motility
**Nucleus**	1	P04083	Annexin 1	38.787/6.64	↓	↑	4.53	Plasm membrane	Calcium binding protein
	2	P07355	Annexin 2	38.677/7.56	↓	↑	3.27	Plasm membrane	Calcium binding protein
	3	P33991	Replication Licensing factor,MCM4	97.068/6.28	↓	↑	1.17	Nucleus	Initiation of DNA replication biosynthesis
	4	NP031478	Peroxiredoxin III	28.047/6.73	↑	↓	0.08	Mitochondria	Antioxidant enzyme
**Membrane**	1	P08670	Vimentin	49.680/5.19	↓	↑	3.57	Cytoplasm	Cell motility
	2	P35232	Prohibitin	29.859/5.57	↑	↓	0.14	Mitochondria	Cell proliferation

**Table 6:** Summary of the differentially expressed proteins in HepG2 and SK-Hep–1 cells using MALDI-TOF MS and categorized according to their functional roles.

Classification	Identified protein	Hep-G2	SK-Hep–1
Group Ⅰ Intermediate filament	Annexin–1	+	+++++ In nucleus
	Annexin–2	-	++++ In nucleus
	Annexin–4	++++ In cytoplasm	+
Group Ⅱ Protection and detoxification	PeroxiredoxinⅠ	++++ In cytoplasm	+
	Peroxiredoxin Ⅱ	++++ In cytoplasm	-
	Peroxiredoxin Ⅲ	+++ In nucleus	-
Group Ⅲ Cytoskeleton protein	Vimentin	-	++++ In membrane
Group Ⅳ Metabolic enzyme	NADP-dependent Isocitrate dehydrogenase	++++ In cytoplasm	-

**Note:** “+” sign denotes positive expression; “-” denotes negative expression

### Differential protein spot analysis

Comparative proteome analysis of various cellular compartments between HepG2 and SK-Hep–1 cells was performed, and 8 protein spots were successfully identified in both cell types. These proteins were classified according to their functional attributes (**Tables 5 and 6**). ANX1 and ANX2 were differentially overexpressed in the nucleus of SK-Hep–1 cells. Conversely, ANX4 was only differentially expressed in the well-differentiated HepG2 cells. Prdx I, II, and III and ICDH were preferentially expressed in the well-differentiated HepG2 cells. Vimentin, an EMT marker, was detected more prominently in SK-Hep–1 cells.

### Confirmation of differential expression by immunofluorescence staining and western blotting

**Fig 3:**
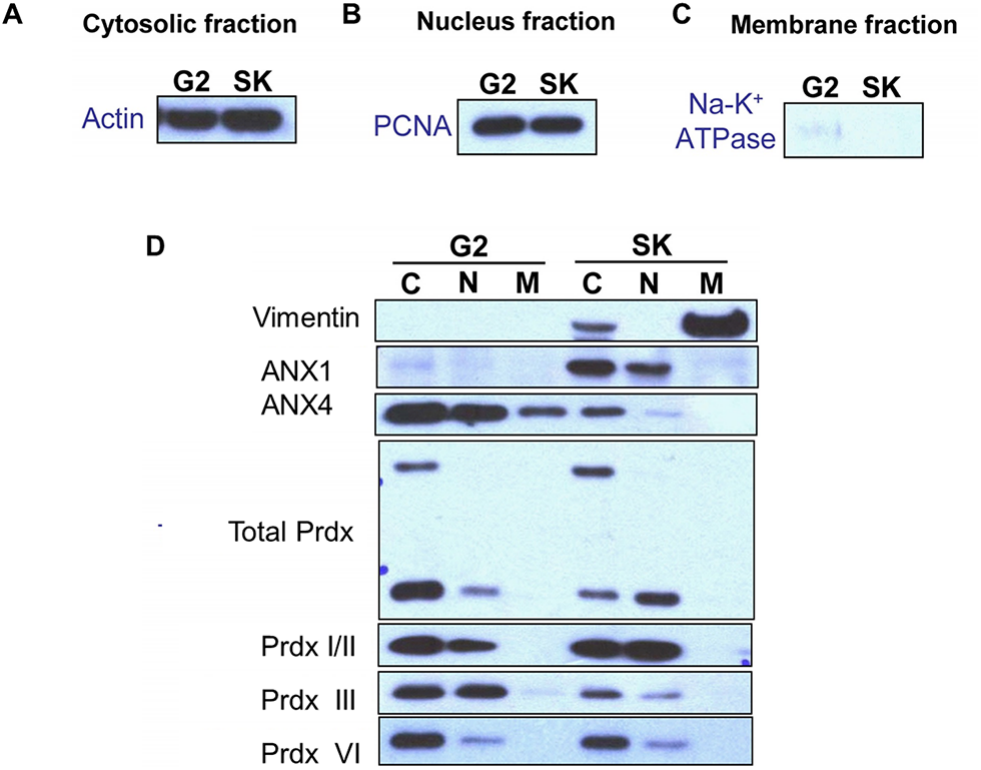
Validation of the differential protein expression between HepG2 and SK-Hep–1 cells by western blot. Actin was used as the internal control in the cytosolic fraction (A), PCNA served as the internal control in the nuclear fraction (B), and Na-K+ ATPase was used as the internal control in the membrane fractions (C). (D) C: cytosolic fraction protein, N: nuclear fraction protein, M: membrane fraction protein.

**Fig 4:**
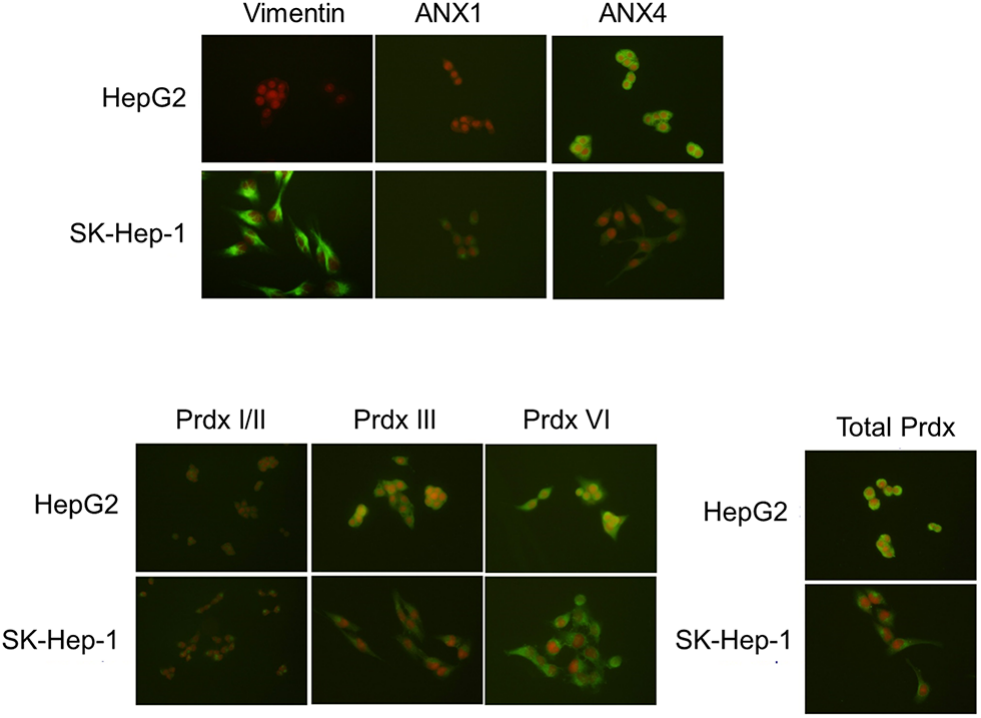
Validation of the differential protein expression between HepG2 and SK-Hep–1 cells by immunofluorescence staining. The expression patterns of ANX1, ANX4 and Prdx were detected by immunofluorescence staining as described in the Methods and Materials.

**Fig 5:**
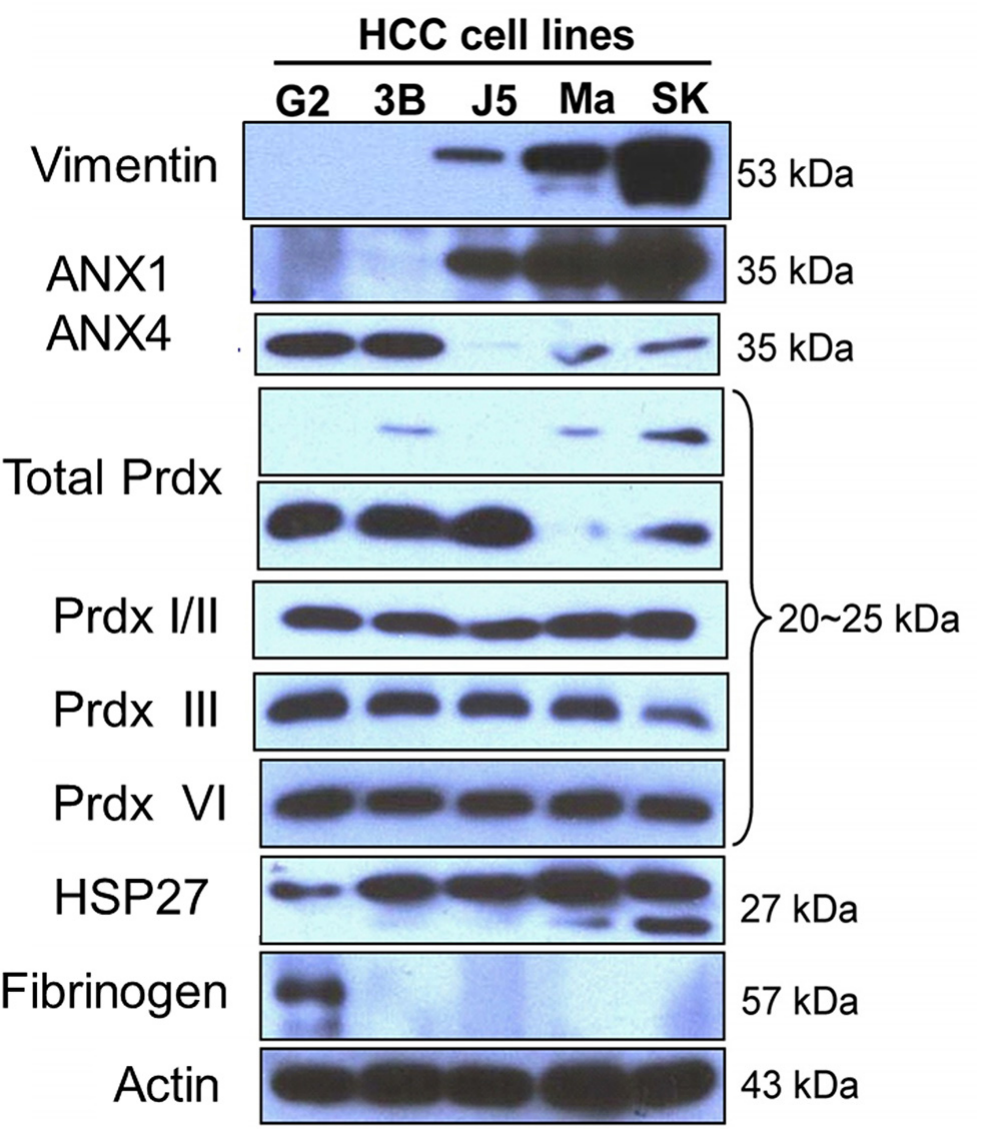
Expression patterns of differentially expressed proteins identified by comparative proteomic analysis in a panel of five HCC sublines with established differentiation/de-differentiation status. The differentiation status (well-differentiated to poorly differentiated) of these five HCC cell lines was on the order of HepG2, Hep3B, HepJ5, Mahlavu, and SK-Hep–1. The expression patterns of ANX1, ANX4, HSP27, Prdx isoforms, vimentin, and fibrinogen were detected by western blotting.

Using actin, PCNA and Na-K^+^ ATPase as internal controls for the cytosolic, nuclear and membrane fractions, respectively, we confirmed the differential expression of the proteins in various cellular compartments by western blotting. As shown in **Fig 3**, ANX4 was highly expressed in the cytosolic and nuclear fractions of the well-differentiated HepG2 cells. In contrast, ANX1 and vimentin were overexpressed in the poorly differentiated SK-Hep–1 cells (**Fig 3**). Immunofluorescence analysis was used to confirm the results of the western blots (**Figs 4 and 5**).

### ANX1 is a biomarker for metastatic potential in HCC cell lines

**Fig 6:**
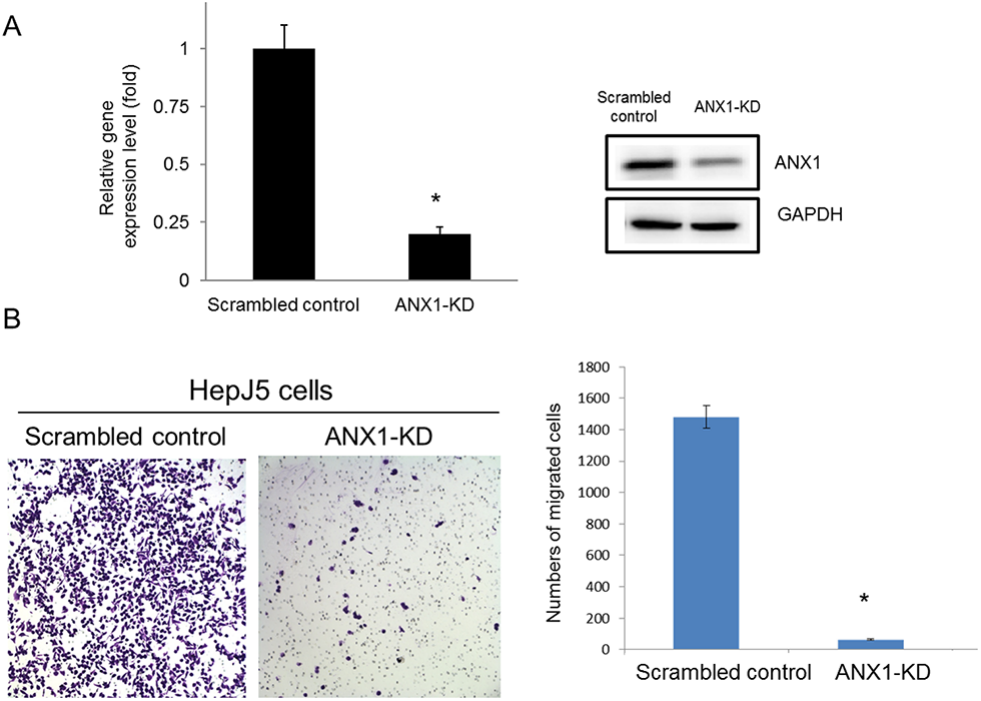
Knockdown of AXN1 expression levels suppressed cell migration. AXN1 expression levels were knocked down by transfection of AXN1-specific shRNA into HepJ5 cells as described in the Methods and Materials. (A) The expression levels of AXN1 in control and AXN1-KD cells were determined by real-time PCR and western blotting. (B) The migration of scrambled control and AXN1-KD cells was determined by the transwell migration assay as described in the Methods and Materials.

The differentially expressed protein candidates identified in HepG2 and SK-Hep–1 cells via our proteomic approach are molecular biomarkers of differentiation status. We validated the expression profiles of these proteins using a panel of HCC cells in a descending order of differentiation based on our previous studies[11, 12]. First, ANX1 expression levels progressively increased as HCC cells became less differentiated (**Fig 6**). The expression profile of vimentin, an EMT marker, was similar to that of ANX1 (**Fig 6**). In contrast, ANX4 was only overexpressed in the well-differentiated HCC sublines, HepG2 and Hep3B. Because vimentin overexpression is generally recognized as a metastatic phenotype indicator in cancer cells, we hypothesized that ANX1 overexpression may serve a similar functional role in promoting metastasis in HCC cells. To test our hypothesis, we silenced ANX1 expression in HepJ5 cells and demonstrated that this manipulation severely impeded the migration of these cells (**Fig 6**). This finding strongly suggests that ANX1 and vimentin are involved in the regulation of the metastatic potential of poorly differentiated HCC cells.

### Higher HSP27 expression is correlated with a highly metastatic potential in HCC cells

**Fig 7:**
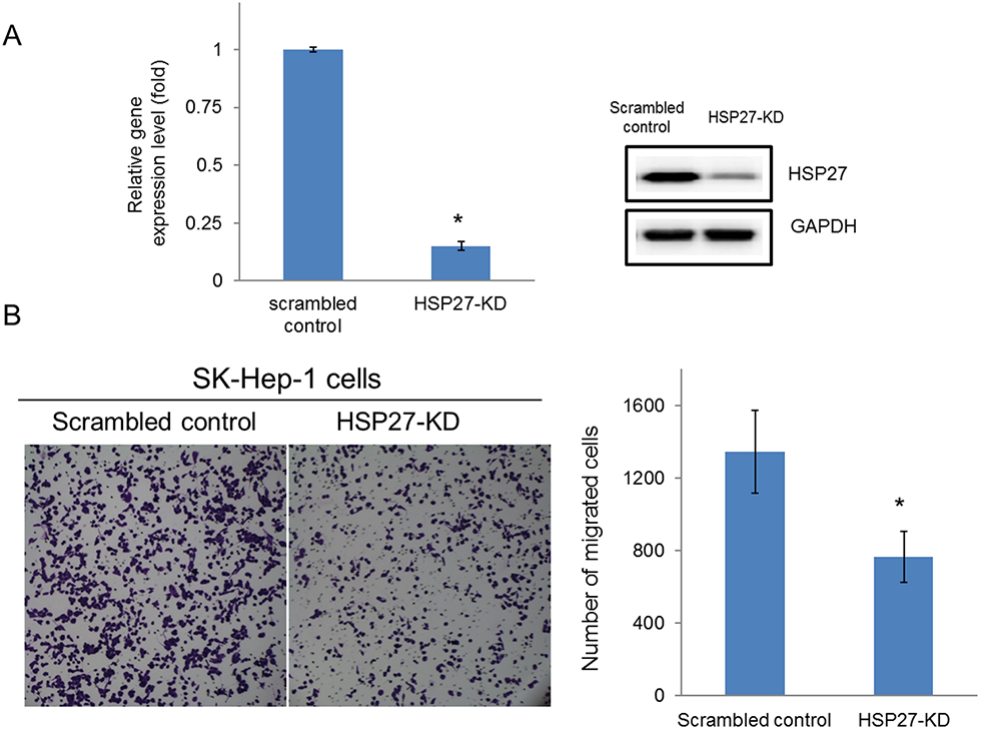
Knockdown of HSP27 expression levels suppressed cell migration. HSP27 expression levels were knocked down by transfection with HSP27-specific shRNA into SK-Hep–1 cells as described in the Methods and Materials. (A) The expression levels of HSP27 in control and HSP27-KD cells were determined by real-time PCR and western blotting. (B) The migration of scrambled control and HSP27-KD cells was determined by transwell migration assayas described in the Methods and Materials.

Consistent with these observations, we also discovered that high expression of HSP27 was correlated with the less differentiated cell types. To confirm the role of HSP27 in HCC cells, we further silenced HSP27 expression by shRNA in SK-Hep–1 cells and performed migration assays. As shown in **Fig 7**, silencing HSP27 dramatically reduced the migration of SK-Hep–1 cells, indicating that HSP27 expression is critical in modulating the differentiation status of HCC cells.

### The pathologic characteristics and expression levels of HSP27 and ANX1

**Fig 8:**
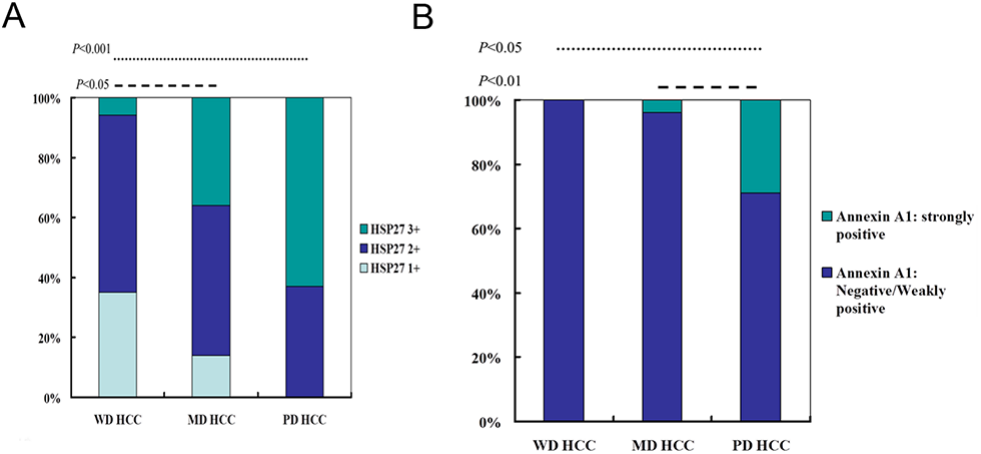
The pathologic characteristics and expression levels of HSP 27 and ANX1. To understand the correlation between pathologic characteristics and expression levels of HSP 27 and ANX1, the immunohistochemical stains with HSP 27 and ANX1 in HCC tissue microarray were performed. HCC was divided into well-differentiated (WD), moderately differentiated (MD) and poorly differentiated (PD). (A) HSP27 expression levels were scored semiquantitatively as weakly positive (1+), moderately positive (2+) and strongly positive (3+). (B) Expression levels of ANX1 were scored semiquantitatively as negative, weakly positive and strongly positive. The association between pathologic characteristics and ANX1 expression was analyzed.

To understand the correlation between pathologic characteristics and expression levels of HSP 27 and ANX1, the immunohistochemical stains with HSP 27 and ANX1 in tissue microarray sets were performed. As shown in **Fig 8,** HCC was divided into well-differentiated (WD), moderately differentiated (MD) and poorly differentiated (PD). HSP27 expression levels were scored semiquantitatively as weakly positive (1+), moderately positive (2+) and strongly positive (3+). PD HCC tended to express stronger HSP27 than WD HCC (*p*<0.001) (**Fig 8A**). MD HCC also revealed stronger HSP27 expression than WD HCC (*p*<0.05). HSP27 expression was not statistically different between PD HCC and MD HCC. In addition, strong ANX1 expression was more commonly found in PD group compared with WD and MD group (*p*<0.05 and *p*<0.01, respectively, **Fig 8B**).

## Discussion

Previous studies have indicated that the levels of reactive oxygen species (ROS) are significantly higher in many cancer cells than in normal cells. Consequently, cancer cells exhibit higher intrinsic oxidative stress [13, 22–25]. The increased ROS stress in cancer cells is associated with de-differentiation and the acquisition of an invasive phenotype [26, 27]. In our study, well-differentiated HCC cells overexpressed antioxidant/metabolic enzymes, such as MnSOD, Prdx, ICDH, α-enolase and UDP-glucose dehydrogenase (**Tables 5 and 6**). In contrast, poorly differentiated HCC cells exhibited high levels of PDI. Therefore, the intrinsic oxidative stress of HCC cells was modulated through the regulation of antioxidant enzymes and was differentiation status-dependent.

Characteristic changes during EMT include the down-regulation of epithelial markers (e.g., E-cadherin) and the up-regulation of mesenchymal markers, such as vimentin and N-cadherin [28–30]. In this study, we initially established that membranous vimentin is abundantly expressed in poorly differentiated SK-Hep–1 cells. In addition, using a panel of five HCC sublines, we demonstrated that vimentin was abundantly expressed in poorly differentiated HCC sublines, including Mahlavu, HepJ5 and SK-Hep–1 cells (**Fig 5**). These data suggest that the de-differentiation of HCC cells is associated with oxidative stress, which may be the driving force and/or protective mechanism for EMT induction and invasive potential. This notion is supported by the up-regulation in many different cancer cell types of NF-κB, which is responsive to stimuli generated during high intrinsic oxidative stress conditions, such as H_2_O_2_ and GSSG [30, 31]. De-differentiated HCC cells, such as the Mahlavu and SK-Hep–1 sublines, exhibited substantial up-regulation of NFκB (data not shown).

The elevated level of ANX1 detected in poorly differentiated HCC cells, such as Mahlavu and SK-Hep–1 cells, attracted our attention. ANX1 belongs to a family of calcium/phospholipid-binding and actin regulatory proteins. Graauw et al.[32]reported that ANX1 and its family member ANX2 are candidate regulators of the oncogene-induced cell morphology switch. During such a switch, tumor cells change from an epithelial to a more migratory, mesenchymal-like phenotype, thus leading to metastasis and the progression of cancer. Our data are consistent with these findings, as the expression pattern of ANX1 was similar to that of vimentin in HCC cells. Increased expression of ANX1 was only observed in poorly differentiated sublines, such as Mahlavu and SK-Hep–1 cells, suggesting that ANX1 is associated with the invasive phenotype (**Fig 5**). Consistently, down-regulation of ANX1 significantly reduced the migration of invasive HepJ5 cells (**Fig 6**) and strong ANX1 expression was more commonly found in PD group compared with WD and MD group (Fig 8B). Those results demonstrates that ANX1 may play a pivotal pole in increasing the metastatic potential of cancer cells and that ANX1 may serve as a biomarker for the aggressive phenotype of HCC cells in conjunction with vimentin.

In contrast to ANX1, ANX4 was uniquely overexpressed in only well-differentiated HCC cells, including the HepG2 and Hep3B sublines. Although the exact role of ANX4 in HCC carcinogenesis remains unclear, Han et al. has suggested that overexpression of ANX4 is associated with paclitaxel resistance in cancer cells [33]. Whether ANX4 can also contribute to the acquisition of chemoresistance in HCC cells warrants further investigation.

Finally, among the differentially expressed proteins, the elevated expression of HSP27 was associated with the less differentiated cellular phenotype (**Fig 5**). Previous studies have indicated that phosphorylation plays a key role in the regulation of HSP27 function and may contribute to the survival of cells during oxidative stress and apoptosis [34, 35]. In addition, HSP27 participates in maintaining GSH in its reduced form during oxidative stress [36]. Another study demonstrated that elevated expression of HSP27 is correlated with the enhanced migration of endothelial cells [37]. Silencing of HSP27 significantly reduced the migration of invasive SK-Hep–1 cells (**Fig 7**). Besides, we also demonstrated that PD HCC tended to expression strong HSP27 than WD HCC (*p*<0.001) (**Fig 8A**). MD HCC also revealed stronger HSP27 expression than WD HCC (*p*<0.05). Collectively, these observations suggest that overexpression of HSP27 in de-differentiated HCC cells may be a molecular indicator for HCC metastatic potential.

In conclusion, we have unveiled a group of biomarkers that are relevant to the metastatic status of HCC cells using a proteomic approach and subsequent validation in a panel of five HCC sublines with varying degrees of differentiation. Collectively, a panel of biomarkers, including vimentin, ANX1 and HSP27, were identified as metastatic indicators for HCC cells in our study and may be considered potential therapeutic targets.
